# Fast-Food Consumption, Dietary Quality, and Dietary Intake of Adolescents in Saudi Arabia

**DOI:** 10.3390/ijerph192215083

**Published:** 2022-11-16

**Authors:** Walaa A. Mumena, Amaal A. Ateek, Rawan K. Alamri, Sarah A. Alobaid, Salwa H. Alshallali, Samah Y. Afifi, Ghaida A. Aljohani, Hebah A. Kutbi

**Affiliations:** 1 Clinical Nutrition Department, College of Applied Medical Sciences, Taibah University, P.O. Box 344, Madinah 42353, Saudi Arabia; 2 Clinical Nutrition Department, Faculty of Applied Medical Sciences, King Abdulaziz University, P.O. Box 80215, Jeddah 21589, Saudi Arabia

**Keywords:** fast-food, dietary quality, dietary intake, predictors, adolescents, Saudi Arabia

## Abstract

High fast-food consumption is a common public-health concern among adolescents, due to its link to a number of non-communicable diseases. Frequent consumption of fast food may also affect diets of individuals; however, research addressing this issue is lacking in Saudi Arabia. We aimed to investigate the association between fast-food consumption, dietary quality, and dietary intake of adolescents in Saudi Arabia. This is a cross-sectional study of 617 healthy adolescents aged 11–18 years, who were recruited randomly from 16 middle- and high-schools located in Jeddah and Madinah, Saudi Arabia. Sociodemographic data were collected from parents. Dietary data, including the frequency of fast-food consumption, dietary quality (assessed using the short-form food frequency questionnaire), and dietary intake (assessed using multiple 24 h diet recalls from a subsample), were collected from the adolescents. Approximately one-third of adolescents (28.5%) reported frequent fast-food consumption (>two times a week). Results showed that a higher proportion of male adolescents frequently consumed fast-food, compared with female adolescents (32.8% vs. 24.8%, *p* = 0.039). Adolescents with the highest monthly family-income (≥SAR 21,000 ) reported a significantly higher frequency of fast-food consumption compared with families with a lower monthly income (*p* = 0.009). Frequency of fast-food consumption predicted lower dietary-quality in adolescents (Beta (B) = −0.27 [95% confidence interval (CI): −0.35 to –0.18]) and higher carbohydrate and free-sugar intake (B = 6.93 [95% CI: 0.78 to 13.1], and B = 3.93 [95% CI: 1.48 to 6.38], respectively). In conclusion, nutrition-intervention programs aiming to limit fast-food consumption and enhance the dietary quality of adolescents in Saudi Arabia, are warranted.

## 1. Introduction

The population of Saudi Arabia has witnessed a significant transformation in eating habits and dietary patterns, due to the rapid economic transition and associated technological and social changes [1,2]. During the past few decades, fast-food has been notably integrated into the Arabian diet, leading to its frequent consumption [3,4]. Fast-food is defined as foods that are purchased from fast-food restaurants, including pizzas, burgers, fried chicken, French fries, and white-flour baked goods [5]. Fast-food is typically energy-dense, rich in saturated fat and salt, and considerably low in several important nutrients [6,7]. Frequent exposure to fast-food has also been found to be negatively associated with fruit, vegetable, fiber, and milk intake [6,8,9,10], and positively associated with the intake of fats, carbohydrates, added sugars, and sugar-sweetened beverages (SSBs) [4,8,9,11].

The findings of studies conducted in several settings suggest that adolescents are the main fast-food consumers [8,12]. This is most likely due to taste preference, the relatively low cost for large portion sizes, and convenience, as well as the wide availability and popularity of fast-food restaurants [13,14,15]. In the United States, the proportion of children and adolescents who consumed fast-food in a given day was 36% [12]. In Saudi Arabia, a study conducted in 2020 indicated a high consumption of fast-food and junk food (87%) [3]. An earlier study conducted among Saudi adolescents showed that around 85% of adolescents prefer to eat fast-food instead of homemade food [16]. 

The increased consumption of fast-food among adolescents poses a significant public health concern, due to its adverse effects on health and well-being. In several studies, fast-food consumption has been linked to increased body mass index (BMI) and insulin resistance, which may lead to an increased risk of obesity and a number of non-communicable diseases, including type 2 diabetes in adulthood [17,18]. In addition, nutritional needs at this age increase significantly to compensate for the rapid physical and physiologic growth, and thus, maintaining proper nutritional status is crucial to prevent micronutrient deficiencies [19].

Despite the available literature on the influence of fast-food consumption on adolescents’ nutritional health, research investigating the association between fast-food consumption and the diet of adolescents in Saudi Arabia is lacking. Thus, we aimed in this study to investigate the association between fast-food consumption, dietary quality, and dietary intake of adolescents in Saudi Arabia. The findings of the present study will inform public-health entities and policy-makers, enabling them to draw evidence-based, culturally tailored recommendations and develop national-level nutrition-interventions to enhance the dietary quality and nutritional status of adolescents.

## 2. Materials and Methods

### 2.1. Study Design and Population

This cross-sectional study included data from healthy adolescents aged between 11 and 18 years old. Healthy adolescents were recruited from middle- and high-schools in Madinah and Jeddah cities, Saudi Arabia. The exclusion criteria included adolescents with chronic diseases, food allergies and dietary restrictions, and adolescents on medication that may affect body weight. The minimum sample size needed for this study was 235 adolescents, based on a mean dietary-quality score of 11.4, a detectable difference of 4% in mean value between the study groups, and a standard deviation of 1.60 [20], 90% power, and alpha (two-sided) = 0.05 [21].

### 2.2. Data Collection

Data were collected from 16 schools between October and November 2021. Schools targeted for the study included one public middle school and another private school for boys and girls (4 schools) and one state high school and another private school for boys and girls (4 schools). Similar criteria for the schools approached in each city was adopted (8 schools in Madinah and 8 schools in Jeddah). 

In each school, 125 students from randomly selected classes received an envelope (2000 envelopes in total were distributed across all schools). Each envelope included a brief description of the study objectives, a parental-consent form for participation, and a questionnaire. Parents were requested to sign the consent form and complete the sociodemographic information, including characteristics of the adolescents and their mothers (adolescents: date of birth, nationality, city of residence, order of child, sex, and food allergy status; mothers: marital status, educational level, employment, working hours, and monthly family-income). Adolescents who returned the envelops with the completed forms and signed consent-forms were proceeded for anthropometric and dietary assessment. All data were collected within two weeks from the date the envelops were initially distributed.

#### 2.2.1. Anthropometric Assessment

The height and weight of all the adolescents were collected objectively in schools by trained healthcare professionals. A measuring tape was used to assess height after it was incorporated into a straight wall. Each student was asked to take off heavy clothes and shoes, stand straight, and look in a 90-degree direction. The height was measured in centimeters (cm) and the measurement was rounded to the nearest 0.5 cm. The weight was measured in kilograms (kg) using an electronic scale (Omron, BF508, Japan) and rounded to the nearest 0.1 kg. Height and weight were measured two times; if the two readings differed by 0.5 cm or 0.1 kg, respectively, a third measurement was taken, and the average of the three measurements was recorded. The recorded measurements were later used to calculate the BMI percentile for each adolescent, based on the Center for Disease Control cutoffs to estimate weight status as follows: underweight (<5th percentile); healthy weight (5th to <85th percentile); overweight (85th to <95th percentile); obese (≥95th percentile) [22].

#### 2.2.2. Assessment of Dietary Quality

The dietary quality of the was assessed using a modified version of the short-form of the food frequency questionnaire (SFFFQ) [20]. The SFFFQ have been used to assess the dietary quality of individuals in different populations [23,24]. Modifications of the tool were made by two experts, to ensure that foods that are typically consumed by the Saudi population were included. The adolescents were guided through the SFFFQ by the research team, and were requested to fill out a hard copy of the questionnaire in class. The SFFFQ included 20 food items: Fruits; Fruit juice; Salad; Cooked vegetables; Fried potatoes/chips; Beans or legumes; Fiber-rich breakfast cereal; Whole wheat bread; Cheese/Yoghurt; Crisps/Savory snacks; Sweet biscuits; Ice cream/cream; Fizzy drinks/Pop; Beef or lamb; Chicken or turkey; Processed meats/meat product; Processed chicken/turkey; Fried white fish; White fish; Oily fish. An Excel sheet was used to enter the frequency of consumption, and the total score for dietary quality was calculated automatically.

#### 2.2.3. Assessment of Dietary Intake

Multiple 24 h diet recalls were collected over the phone from a subsample of 34.8% (n = 258); three 24 h diet recalls (two weekdays and one weekend day) were collected from 94.6% of the subsample (n = 244), whereas two 24 h diet recalls were collected from the remaining 5.40% of the subsample (n = 14). Participants were asked by trained data-collection personnel to remember all food items consumed in the last 24 h. Images of standardized measurements were sent via WhatsApp to participants to further assist in estimating the quantities of food consumed. Data concerning type and quantities of food consumed were recorded on paper, in order to be entered later into the software. The Nutritics software was used to analyze dietary data (version 5.09, Dublin, Ireland), and has been used previously [25,26]. The database of Nutritics software includes several Middle Eastern food recipes. When a specific food recipe was not found, ingredients were inserted into the system manually, based on a standardized recipe. Four experts in the field of nutritional assessment provided all of the required training sessions, and two of them supervised the collection and entry of the dietary data. 

#### 2.2.4. Assessment of Fast-Food Consumption

The adolescents were asked to report the frequency of fast-food consumption in a typical week (a continuous variable). The question was explained to the adolescents by defining fast-food as food purchased from fast-food restaurants including pizza, burger, French fries, fried chicken, shawarma, and hotdogs. The reported data were later collapsed into two categories, to identify non-frequent and frequent fast-food consumers as follows: (1) ≤2 times per week: non-frequent fast-food consumers; (2) >2 times per week: frequent fast-food consumers [27,28].

### 2.3. Statistical Analysis

Data collected in the study were analyzed using the Statistical Package for the Social Sciences (SPSS) (IBM Corp. Released 2017. IBM SPSS Statistics for Windows, Version 25.0. Armonk, NY, USA: IBM Corp). Data were presented as mean ± standard deviation (SD) and median (interquartile range). Fisher’s exact test was used to assess the significance across two categorical variables. For continues variables, the normality of all distributions (dietary-quality score and nutrient intake) was assessed using the Shapiro–Wilk test. To compare the median dietary-quality score among the groups of fast-food consumption (frequent vs. non-frequent), the Mann–Whitney test was used. The Bonferroni adjustment for multiple testing was used to adjust the alpha in the univariate analysis. Multiple linear regression analysis was used to investigate the association between fast-food consumption (predictor) and dietary-quality score and nutrient intake (outcomes); models were adjusted for the student’s age, sex, family income, and city of residence. Categorical variables used in the regression models were coded as follows: sex (male = 1; female = 2), city of residence (Jeddah = 0; Madinah = 1), monthly family income in Saudi Riyal (SAR) (<SAR 6000 = 1; SAR 6000–10,999 = 2; SAR 11,000–15,999 = 3; SAR 16,000–20,999 = 4; ≥SAR 21,000 = 5). Tests used were two-tailed, at a significance level of 95%.3.

## 3. Results

### 3.1. Sample Characteristics

A total of 741 envelopes were returned with signed consent-forms (a 37.1% response rate). We excluded 9.31% (n = 69) of the sample due to missing data (weight status, dietary data, fast-food consumption); 4.59% (n = 34) due to food allergies; 2.16% (n = 16) due to chronic disease; 0.40% (n = 3) due to dietary restriction; 0.27% (n = 2) were older than 18 years of age. The final analysis included data from 617 adolescents. Approximately one-third of the sample (28.5%, n = 176) were identified as frequent fast-food consumers (>two times a week).

Table 1 represents characteristics of the sample stratified by frequency of fast-food consumption. A significantly higher proportion of males were frequent fast-food consumers, compared with female adolescents (32.8% vs. 24.8%, *p* = 0.039). Additionally, a significantly higher proportion of frequent fast-food consumers had a monthly income of ≥SAR 21,000, as compared to those with a lower monthly family-income (*p* = 0.009). All other sociodemographic characteristics were similar across the study groups (fast-food consumption ≤ 2 times a week vs. fast-food consumption > 2 times a week).

### 3.2. Association of Fast-Food Consumption with Dietary Quality and Dietary Intake

The median dietary-quality score of adolescents who reported a frequent fast-food consumption of > 2 times a week was significantly lower, compared with the median dietary-quality score of adolescents who consumed fast-food ≤ 2 times a week (9.00 (7.00–10.0) vs. 10.0 (9.00–11.0), respectively, *p* < 0.001) (see Table 2). In addition, frequent fast-food consumers had a significantly higher energy-intake (1371 (1104–1826) kilocalories (kcal)) compared with non-frequent fast-food consumers (1179 (957–1552) kcal), *p* = 0.002. Furthermore, the frequent fast-food consumers had a significantly higher carbohydrate-intake (175 (135–219) grams (g)) compared with non-frequent fast-food consumers (140 (113–185) g), *p* < 0.001). The intake of all other nutrients was similar across the different groups of fast-food consumption. 

A multiple linear regression analysis of dietary quality and dietary intake in relation to fast-food consumption was performed, adjusting for age, sex, family income, and city of residence. The frequency of fast-food consumption predicted a lower dietary-quality (beta (B) = −0.27, standard error (SE) = 0.04 [95% confidence interval (CI): −0.35 to −0.18], R-square = 0.09), (see Table 3). Furthermore, the frequency of fast-food consumption predicted a higher carbohydrate and free-sugar intake (B = 6.93, SE = 3.12 [95% CI: 0.78–13.1], R-square = 0.14 and B = 3.93, SE = 1.24 [95% CI: 1.48–6.38], R-square = 0.10, respectively). Fast-food consumption did not predict the intake of any other nutrients.

## 4. Discussion

Approximately one-third of the adolescents included in this study reported frequent fast-food consumption (>two times a week). A higher proportion of male adolescents frequently consumed fast-food, compared with female adolescents. Adolescents with the highest monthly family-income reported a higher frequency of fast-food consumption, compared with families with a lower monthly income. The dietary-quality score was significantly lower among frequent fast-food consumers, while a higher intake of carbohydrate and free sugar was observed among adolescents who frequently consumed fast-food. 

The findings of the current study show that a higher intake of fast-food significantly predicts lower dietary quality and a higher intake of free sugar among adolescents. This is consistent with previous findings suggesting that individuals who frequently consume fast-food tend to consume lower-quality diets that contain more free sugar and less fiber, milk, fruit, and fewer vegetables [6,8,9,10,11]. This association was expected, as fast-food is typically consumed with SSBs, and most adolescents prefer to drink soda instead of water, fresh juice, or milk, with meals. In fact, a high intake of free sugar has been found to be independently associated with a low intake of several important nutrients such as calcium, zinc, potassium, sodium, and vitamin B12 [29]. Meanwhile, a high intake of fast-food, as well as free sugar, has been linked to an increased risk of multiple health issues, such as obesity, cardiovascular diseases, insulin resistance, diabetes, and mortality [17,18,30,31].

The present study suggests that adolescents who consume fast-food > 2 times a week have a higher consumption of carbohydrate, compared with non-frequent consumers of fast-food. This is most likely due to the high consumption of carbohydrate-rich foods, such as SSBs, French fries, high-sugar sauces, and bread [8,16]. In recent studies, fast-food consumption was associated with the consumption of larger meals, leading to an increased carbohydrate intake [32]. Additionally, fast-food meal-items are considered as high glycemic-index foods, which are known to affect metabolic and hormonal responses leading to increased appetite, overeating, and rapid return to feeling hungry; therefore, fast-food consumption may result in an increase of overall food-intake during the day [33,34]. On the other hand, limiting the consumption of fast-food may decrease the intake of simple sugars and potentially decrease obesity and obesity-related non-communicable diseases [35]. As such, public-health-education programs are needed, to raise awareness concerning healthy food options, in order to promote a healthy lifestyle and dietary habits among the Saudi population [36]. A healthy home- and school-food environment may also facilitate the adoption of healthy dietary habits, and enhance the diet quality of adolescents [37]. Students may benefit from school meal programs that offer healthy food options while limiting the availability of low-quality foods sold in canteens, such as chips, candies, juices, milk, croissants and biscuits. Additionally, interactive nutrition-education programs delivered through social media may also promote healthy dietary habits among adolescents [38]. 

In the current study, the sex of the adolescents was associated with fast-food consumption; a significantly larger proportion of males frequently consumed fast food, compared with females. A study conducted among 1133 adolescents in the Qassim region, Saudi Arabia, showed that female adolescents are less likely to consume fast-foods, compared with male adolescents [39]. This association was also found among university students in Saudi Arabia, and in other settings [40,41]. We postulate that females care more about their appearance, so they tend to have a higher awareness-level related to healthy foods, and they tend also to choose smaller portion sizes for their meals, compared with males [42]. Additionally, global data show that males tend to cook less frequently, compared with females [43], and thus, fast-food might be a more accessible option, especially for late dinners. A descriptive study conducted in 2020 among the Saudi population found that 79% of fast-food is consumed at dinner [3]. 

Monthly family-income in the present study was found to be associated with fast-food consumption; the greatest proportion of frequent fast-food consumers were those with the highest monthly family-income (≥SR 21,000). A nationwide survey conducted in Australia found that the intake of fast-food has been greater among people with high income levels [44]. Another study conducted in the United States showed that children and adolescents from lower-income families reported less frequent fast-food consumption, compared with children and adolescents from higher-income families [15]. Higher accessibility of fast-food can explain the higher frequency of consumption among high-income individuals.

This is the first study to investigate the association between fast-food consumption and the diet of adolescents in Saudi Arabia. In addition, the study sample was recruited randomly from state and private schools, to increase the external validity of the study findings. However, the cross-sectional design used in this study did not allow for the determination of causality between fast-food consumption and dietary quality/intake. In addition, the school- and home-environment and factors related to time of meals which may influence the link between fast-food intake and the diet of adolescents, were not explored.

## 5. Conclusions

Fast-food is frequently consumed by adolescents in Saudi Arabia, especially males and children with a high monthly family-income. Frequent fast-food consumption was found to be associated with lower dietary-quality and a higher intake of carbohydrates and free sugar. The findings of this study highlight the urgency of developing intervention programs and policies that aim to limit the consumption of fast-food among adolescents in Saudi Arabia. More research is needed to investigate the association between fast-food consumption and dietary quality among other age groups in Saudi Arabia.

## Figures and Tables

**Table 1:** Characteristics of participants stratified by frequency of fast-food consumption (n = 617).

Variable	Fast-Food Consumption ≤ 2 Times a Week (n = 441)	Fast-Food Consumption > 2 Times a Week (n = 176)	*p-*Value
Adolescents			
Age group			
12–14 years	202 (71.1)	82 (28.9)	0.858
15–18 years	239 (71.8)	94 (28.2)	
Sex			
Males	195 (67.2)	95 (32.8)	0.039 ^1^
Females	246 (75.2)	81 (24.8)	
Nationality			
Saudi	357 (69.9)	154 (30.1)	0.059
Non-Saudi	84 (79.2)	22 (20.8)	
City of residence			
Jeddah	157 (72.7)	59 (27.3)	0.576
Madinah	284 (70.8)	117 (29.2)	
School			
Private	188 (68.9)	85(31.1)	0.243
State	253 (73.5)	91 (26.5)	
Order of child			
Older child	129 (75.9)	41 (24.1)	0.513
Middle child	200 (69.9)	86 (30.1)	
Youngest child	104 (69.8)	45 (30.2)	
Only child	8 (66.7)	4 (33.3)	
Weight status			
Underweight	43 (72.9)	16 (27.1)	0.544
Healthy weight	231 (69.2)	103 (30.8)	
Overweight	72 (73.5)	26 (26.5)	
Obese	95 (75.4)	31 (24.6)	
Mothers			
Age group			
19–40 years	196 (74.5)	67 (25.5)	0.245
41–50 years	191 (68.0)	90 (32.0)	
>50 years	54 (74.0)	19 (26.0)	
Marital status			
Married	399 (71.0)	163 (29.0)	0.283
Separated	26 (70.3)	11 (29.7)	
Widow	16 (88.9)	2 (11.1)	
Education level			
≤High school	181 (75.4)	59 (24.6)	0.236
University degree	218 (69.2)	97 (30.8)	
Postgraduate degree	42 (67.7)	20 (32.3)	
Employment			
Unemployed	272 (73.5)	98 (26.5)	0.326
Employed	148 (67.6)	71 (32.4)	
Retired	21 (75.0)	7 (25.0)	
Working hours per day			
<4 h	2 (50.0)	2 (50.0)	0.087
4–6 h	50 (69.4)	22 (30.6)	
7–8 h	85 (70.2)	36 (29.8)	
>8 h	10 (47.6)	11 (52.4)	
N/A	294 (73.7)	105 (26.3)	
Monthly family-income			
<SAR 6000	110 (82.7)	23 (17.3)	0.009 ^1^
SAR 6000–10,999	102 (69.9)	44 (30.1)	
SAR 11,000–15,999	79 (66.4)	40 (33.6)	
SAR 16,000–20,999	80 (73.4)	29 (26.6)	
≥SAR 21,000	70 (63.6)	40 (36.4)	

^1^ Alpha = 0.05. SAR: Saudi Riyal ($ 1 = SAR 3.75).

**Table 2:** Association between fast-food consumption, dietary quality, and dietary intake of adolescents in Saudi Arabia.

	Fast-Food Consumption ≤ 2 Times a Week (n = 441)	Fast-Food Consumption > 2 Times a Week (n = 176)	*p-*Value
Dietary-quality score	9.53 ± 1.68	8.56 ± 1.70	<0.001 ^1^
	10.0 (9.00–11.0)	9.00 (7.00–10.0)	
Energy, kcal	1318 ± 665	1515 ± 585	0.002 ^1^
	1179 (957–1552)	1371 (1104–1826)	
Carbohydrate, g	155 ± 67.9	186 ± 70.3	<0.001 ^1^
	140 (113–185)	175 (135–219)	
Protein, g	52.2 ± 44.9	56.6 ± 26.2	0.047
	45.1 (32.8–60.5)	48.7 (36.1–70.3)	
Fat, g	51.9 ± 32.7	57.9 ± 27.0	0.036
	44.7 (34.2–61.8)	51.2 (38.7–77.3)	
Fiber, g	9.57 ± 4.69	10.6 ± 4.98	0.063
	8.26 (6.37–11.8)	9.63 (7.47–12.9)	
Free sugar, g	38.9 ± 25.0	49.9 ± 30.6	0.004
	34.1 (21.4–50.7)	44.3 (26.3–67.7)	
Sodium, mg	2079 ± 1359	2867 ± 3479	0.004
	1719 (1234–2477)	2139 (1616–2727)	
Potassium, mg	1409 ± 1081	1522 ± 707	0.058
	1250 (949–1629)	1393 (996–1854)	
Calcium, mg	439 ± 282	432 ± 197	0.643
	392 (270–517)	379 (305–513)	
Phosphorus, mg	652 ± 409	692 ± 285	0.107
	589 (435–772)	644 (492–834)	
Manganese, mg	1.53 ± 0.87	1.59 ± 0.76	0.239
	1.29 (0.98–1.94)	1.41 (1.16–1.97)	
Iron, mg	8.82 ± 5.34	9.80 ± 4.38	0.011
	7.88 (5.58–10.2)	8.93 (6.97–11.5)	
Zinc, mg	5.03 ± 6.23	4.97 ± 2.16	0.117
	4.00 (2.96–5.62)	4.57 (3.20–6.30)	
Vitamin D, mg	11.4 ± 54.5	9.69 ± 21.9	0.689
	1.64 (0.53–3.81)	1.61 (0.46–2.98)	
Vitamin B_12_, mg	2.04 ± 3.23	1.96 ± 1.50	0.203
	1.44 (0.79–2.30)	1.55 (1.00–2.56)	
Vitamin C, mg	44.9 ± 57.9	40.7 ± 35.9	0.647
	34.2 (16.1–56.3)	29.9 (16.9–53.6)	

^1^ Alpha = 0.003 after Bonferroni adjustment.

**Table 3:** Multiple linear regression analysis of dietary quality and dietary intake in relation to fast-food consumption among adolescents in Saudi Arabia.

	Beta	Standard Error	95% Confidence Interval	*p-*Value	R-Square
Dietary-quality score	−0.27	0.04	−0.35 to −0.18	<0.001 ^1^	0.09
Energy, kcal	37.2	29.1	−20.2 to 94.6	0.203	0.13
Carbohydrate, g	6.93	3.12	0.78 to 13.1	0.027 ^1^	0.14
Protein, g	−0.24	1.81	−3.81 to 3.33	0.894	0.12
Fat, g	1.28	1.44	−1.57 to 4.12	0.377	0.07
Fiber, g	−0.14	0.22	−0.47 to 0.39	0.862	0.10
Free sugar, g	3.93	1.24	1.48 to 6.38	0.002 ^1^	0.10
Sodium, mg	171	106	−38.1 to 379	0.109	0.04
Potassium, mg	−0.81	45.3	−90.1 to 88.4	0.986	0.08
Calcium, mg	−6.33	12.4	−30.7 to 18.0	0.610	0.02
Phosphorus, mg	−6.02	17.1	−39.6 to 27.6	0.725	0.11
Manganese, mg	−0.05	0.04	−0.13 to 0.02	0.153	0.11
Iron, mg	−0.001	0.24	−0.47 to 0.47	0.998	0.04
Zinc, mg	−0.24	0.25	−0.74 to 0.25	0.338	0.05
Vitamin D, mg	−1.81	2.23	−6.20 to 2.57	0.416	0.03
Vitamin B12, mg	−0.08	0.13	−0.34 to 0.19	0.571	0.04
Vitamin C, mg	−2.12	2.49	−7.04 to 2.79	0.396	0.01

^1^ Alpha = 0.05. All models were adjusted for age, sex, family income, and city of residence.

## Data Availability

The data that support the findings of this study are available from the corresponding author upon reasonable request.
